# Comparison of Reoperation and Complication Rates Between Acute and Delayed Advanced Nerve Interface Procedures in Lower-Extremity Amputees

**DOI:** 10.3390/jcm15020882

**Published:** 2026-01-21

**Authors:** Kevin Kuan-I Lee, Omer Sadeh, Alberto Barrientos, Anne Genzelev, Omri Ayalon, Nikhil A. Agrawal, Jonathan M. Bekisz, Jacques H. Hacquebord

**Affiliations:** 1 Division of Hand Surgery, Department of Orthopedic Surgery, NYU Langone Health, New York, NY 10016, USA; 2 Hansjörg Wyss Department of Plastic Surgery, NYU Langone Health, New York, NY 10016, USA

**Keywords:** targeted muscle reinnervation, regenerative peripheral nerve interface, lower-extremity amputation, surgical timing, postoperative complications

## Abstract

**Background/Objectives**: Targeted muscle reinnervation and regenerative peripheral nerve interface procedures have emerged as effective techniques for reducing post-amputation pain and preventing symptomatic neuroma formation. However, the optimal timing of these procedures remains debated. This study aims to compare complication and reoperation rates between acute and delayed advanced nerve interface procedures in lower-extremity amputees. **Methods**: A retrospective cohort study was conducted including 74 patients who underwent acute or delayed targeted muscle reinnervation and/or regenerative peripheral nerve interface procedures between 2019 and 2025 at a tertiary academic medical center. Procedures performed concurrently with amputation or during early-stage reconstruction were classified as acute, whereas procedures performed more than one month after amputation were classified as delayed interventions. The primary outcome was postoperative surgical complications occurring within one year. Mann–Whitney U and chi-square tests were used for group comparisons. Univariable and multivariable logistic regression analyses were performed to identify factors associated with surgical complications, adjusting for potential confounders. A *p*-value < 0.05 was considered statistically significant. **Results**: Of 80 limbs, 47 (58.8%) underwent acute and 33 (41.3%) underwent delayed procedures. One-year complication rates were 23.4% in the acute group, and 12.1% in the delayed group, with wound-related complications predominantly occurring in patients undergoing amputation for infection or vascular disease. Unexpected reoperation rates were 19.1% for acute and 12.1% for delayed interventions. On univariable and multivariable analyses, early procedures demonstrated higher odds of surgical complications. However, these associations did not reach statistical significance and were limited by baseline differences in patient comorbidity and etiology. **Conclusions**: Early advanced nerve interface procedures were performed in more medically complex patients and were associated with higher observed rates of surgical complications, whereas delayed procedures were associated with a higher incidence of recurrent symptomatic neuromas. These findings underscore the importance of patient selection, etiology of amputation, and surgical context, rather than timing alone, when determining the optimal approach to nerve interface reconstruction following lower-extremity amputation.

## 1. Introduction

An estimated 185,000 limb amputations are performed in the United States each year [1]. Chronic post-amputation pain is highly prevalent among individuals living with limb loss, significantly diminishing quality of life and contributing to substantial psychological and social distress [2,3]. This debilitating pain can also interfere with effective prosthetic use, further reducing the functional ability of these patients. A large proportion of patients experience both residual limb pain and phantom limb pain, with reported prevalence rates of 76% and 85%, respectively [3]. Much of this chronic pain is associated with, or exacerbated by, symptomatic neuromas that develop at the ends of transected nerves [3,4].

Conventional nerve management techniques after amputation have included traction neurectomy, nerve capping (using synthetic or biological conduits to enclose the nerve end), and burial of free nerve ends into adjacent muscle or bone [5,6]. However, these methods often leave the nerve endings inadequately protected, predisposing them to neuroma formation. Consequently, patients are at increased risk of developing symptomatic neuromas, which can lead to chronic pain and a diminished quality of life. Although originally not intended for neuroma prevention, advanced nerve interface procedures, specifically targeted muscle reinnervation (TMR) and regenerative peripheral nerve interfaces (RPNIs), have been shown to effectively prevent neuroma formation and reduce chronic pain [7,8]. Over the past decade, these techniques have emerged as mainstream surgical approaches for optimizing post-amputation outcomes [9,10,11].

Although there is substantial evidence supporting the clinical effectiveness of these two procedures, discussions regarding the optimal timing of each remain controversial. Previous literature has described their application either prophylactically at the time of amputation (acute) or months to years later in patients with refractory symptoms (delayed) [9,12,13,14]. Recent studies suggest that acute TMR may result in reduced rates of recurrent neuroma formation and could be preferred over delayed intervention after patients become symptomatic [3]. Many existing studies have focused on quantifying functional outcomes, pain reduction, and prosthetic performance following these procedures [15,16]. However, within the literature there is a relative absence of data pertaining to have specifically evaluated complication and reoperation rate, factors that are critical for surgical planning and must be individualized for each patient.

This study aimed to compare complication and reoperation rates associated with acute and delayed TMR and RPNI procedures in lower-extremity amputees. We sought to evaluate whether the timing of nerve interface reconstruction, within the context of differing patient characteristics and amputation etiologies, was associated with differences in postoperative complications and reoperations.

## 2. Materials and Methods

This retrospective cohort study was conducted at a tertiary academic medical center in the United States. The study included a total of 74 patients who underwent 80 lower-extremity amputations with either acute or delayed advanced nerve interface surgery between 2019 and 2025. Since several patients underwent bilateral or multiple staged procedures, each limb was analyzed as an independent unit for outcome measurements. All amputations included in this study were major lower-extremity amputations, defined as transtibial (below-knee) or transfemoral (above-knee) procedures. Eligible patients were 18 years or older and had undergone TMR, RPNIs, or both, with a minimum follow-up period of six months. Patients who were lost to follow-up or lacked adequate postoperative data were excluded. The study protocol was approved by the institutional ethics committee, and all participants provided written informed consent for the publication of clinical data, in accordance with the Declaration of Helsinki on biomedical research involving human subjects.

Data were retrospectively extracted from electronic medical records, including sex, age, body mass index, comorbidities, smoking history, etiology of amputation, amputation level, and the number of nerves addressed. Vascular status was assessed based on the documented etiology of amputation and comorbid diagnoses, including ischemia and peripheral vascular disease. Patients were categorized based on the timing of TMR or RPNIs relative to their primary amputation. Those who underwent nerve interface surgery simultaneously or within 30 days of the amputation were classified as the acute group, whereas those who underwent surgery more than 30 days after amputation were classified as the delayed group. All procedures were performed by attending surgeons specializing in orthopedic surgery and plastic and reconstructive surgery.

For TMR procedures, residual sensory or motor nerves were coapted to adjacent motor nerve branches within nearby target muscles. In RPNI procedures, transected nerve ends were implanted into free autologous muscle grafts [7,17]. The selection of surgical technique was based on the surgeon’s preference and individual patient characteristics. Operative reports were reviewed to document the mechanism of injury and to identify the specific nerves that were transferred or buried.

The outcomes were postoperative complications and reoperation rates occurring within one year following nerve interface surgery. Complications included wound infection, hematoma, non-healing wounds, scar contracture, or recurrent neuroma formation. Reoperations were defined as unplanned surgical interventions related to the initial procedure. All available follow-up records within the one-year period were thoroughly reviewed to capture these events. Although ten limbs did not complete the full one-year follow-up, all had at least six months of postoperative evaluation.

Continuous variables were presented as median values with interquartile ranges (IQR, 25th–75th percentile) and analyzed using the Mann–Whitney U test. Categorical variables were expressed as counts and percentages and compared using the chi-square test. A *p*-value < 0.05 was considered statistically significant. Univariable logistic regression was first performed to evaluate the association between each individual predictor and the presence of surgical complications. Variables with clinical relevance or evidence of association on univariable analysis were then included in a multivariable logistic regression model to adjust for potential confounding factors, including age, sex, body mass index, smoking status, hypertension, dyslipidemia, and peripheral vascular disease. All statistical analyses were performed using MedCalc (Version 20.218; MedCalc Software, Ostend, Belgium).

## 3. Results

We analyzed the demographic and clinical characteristics of the included lower-extremity amputees. A total of 80 lower limbs from 74 patients treated between 2019 and 2025 were analyzed. All included cases were major lower-extremity amputations, consisting of transtibial (below-knee) and transfemoral (above-knee) procedures. Of these, 47 limbs (58.8%) underwent acute TMR/RPNI procedures, while 33 limbs (41.3%) underwent delayed procedures. Among patients with acute amputation, 25 limbs underwent a two-stage procedure, initial guillotine amputation followed by delayed wound closure, while 22 limbs underwent a one-stage amputation. There were no statistically significant differences between the acute and delayed groups with respect to age, sex, body mass index, smoking history, or number of nerves addressed. However, significant differences were observed in comorbidities, particularly hypertension and diabetes mellitus, as well as in the etiology and level of amputation. Patients in the acute cohort were more likely to have a history of hypertension (*p* = 0.026), diabetes mellitus (*p* = 0.026), infection as the etiology of amputation (*p* = 0.008), and a higher proportion of below-knee amputations (*p* = 0.0002) (Table 1).

Among the 47 limbs in the acute group, 61.7% (*n* = 29) underwent TMR alone, 10.6% (*n* = 5) underwent RPNIs alone, and 27.7% (*n* = 13) received both procedures. In contrast, among the 33 limbs in the delayed group, 60.6% (*n* = 20) underwent TMR alone, 27.3% (*n* = 9) underwent RPNIs alone, and 12.1% (*n* = 4) underwent both. Although the distribution of procedures differed between the groups, the difference was not statistically significant (*p* = 0.072) (Table 2).

The overall one-year complication rate was 23.4% (*n* = 11) in the acute group and 12.1% (*n* = 4) in the delayed group (*p* = 0.206). While the overall rates were not significantly different, the types of complications varied between groups. In the acute group, one patient developed a hematoma, three experienced surgical site infections, and seven had non-healing wounds. Notably, none of the patients in this group went on to develop a painful neuroma that needed surgical treatment within one year of surgery. In contrast, in the delayed group, two patients developed postoperative wound infections, and two developed recurrent neuromas that required secondary surgical intervention (Table 3).

When complication rates were stratified by amputation etiology, wound-related complications were more frequently observed among limbs amputated for infection or vascular disease compared with trauma-related amputations across both timing groups. In the acute cohort, complications were more commonly seen in patients with infectious or vascular etiologies, whereas trauma-related amputations demonstrated lower complication rates. A similar pattern was observed in the delayed cohort, although overall complication rates seemed lower than the acute cohort (Table 4).

The unplanned reoperation rate within one year of surgery was 19.1% (*n* = 9) in the acute group and 12.1% (*n* = 4) in the delayed group (*p* = 0.405). Two patients in the acute group and three in the delayed group underwent staged osseointegration for prosthetic fitting, which was not considered an unexpected event. Two patients with wound infections were successfully managed with antibiotic therapy and did not require reoperation. In the acute group, one patient developed a hematoma, one experienced a wound infection, and seven had non-healing wounds, all of which required reoperation. In the delayed group, two patients developed wound infections, and two experienced recurrent neuromas that necessitated surgical revision (Table 5).

Although the surgery-related complication rate was 23.4% (*n* = 11) in the acute group and 6.1% (*n* = 2) in the delayed group, univariable logistic regression demonstrated higher odds of surgical complications in the acute cohort compared with delayed interventions, though this did not reach statistical significance (OR 4.74, 95% CI 0.97–23.02; *p* = 0.054). In the multivariable logistic regression model adjusting for age, sex, BMI, smoking status, hypertension, dyslipidemia, and peripheral vascular disease, acute procedures continued to demonstrate higher estimated odds of postoperative complications, though these findings were limited by wide confidence intervals and did not achieve statistical significance (adjusted OR 4.32, 95% CI 0.73–25.75; *p* = 0.108) (Table 6 and Table 7). The relatively small number of complication events and baseline differences between cohorts should be taken into consideration when interpreting these regression.

## 4. Discussion

The timing of advanced nerve interface reconstruction following lower-extremity amputation remains an area of ongoing clinical debate. This study evaluated complication and reoperation outcomes following acute versus delayed TMR/RPNIs in lower-extremity amputees, with particular attention to the influence of patient selection and surgical context. While higher observed rates of surgical complications were noted among patients undergoing acute TMR/RPNIs, these procedures were typically performed in more medically complex patients and in higher-risk wound environments. In contrast, delayed procedures demonstrated a greater incidence of recurrent neuroma formation requiring revision surgery. These findings align with prior literature indicating that delayed TMR is more frequently performed in patients who are already symptomatic neuroma formers, a population inherently predisposed to neuroma recurrence [3].

TMR and RPNI procedures have demonstrated significant benefits for amputees, including reductions in phantom limb pain and residual limb pain, as well as improvements in mobility and prosthetic function [10,11,18]. A growing body of evidence suggested that performing acute TMR at the time of amputation was a decreased rate of symptomatic neuroma formation and better pain outcomes [16,19,20]. Despite these promising findings, few studies have focused on the complication and reoperation rates of these advanced nerve interface procedures. Compared with amputation alone, whether the addition of TMR increases complication rates remains controversial. Some studies have reported a trend toward a higher incidence of complications [21,22], whereas others have demonstrated lower complication rates [18]. Notably, only a limited number of studies have examined how the timing of surgery may influence complication rates.

Recent evidence has indicated that the complication and reoperation rates are generally comparable, regardless of the timing of nerve interface surgery [3,16]. In contrast, we observed higher rates of surgery-related complications among patients undergoing acute procedures; however, these findings likely reflect differences in patient selection, wound environment, and overall clinical context at the time of surgery, rather than surgical timing alone. Moreover, prior studies often included mixed cohorts of upper and lower-extremity amputees, potentially masking population-specific differences in anatomy, soft-tissue coverage, and biomechanical demands. In contrast, our study focuses exclusively on lower-extremity amputees, providing a more homogeneous and anatomically consistent assessment of outcomes that better reflects the unique challenges in this patient population.

Surgical complications are well recognized to increase both patient mortality and hospital length of stay, leading to prolonged recovery and delayed rehabilitation [23,24]. Beyond their clinical impact, these complications also contribute to a substantial economic burden, not only through extended inpatient care, additional surgical interventions, and increased use of healthcare resources, but also through indirect costs such as lost productivity and long-term disability [25,26]. The financial implications are particularly significant in amputation care, where even minor wound complications can delay prosthetic fitting, extend rehabilitation, and limit functional recovery.

Furthermore, patients who experience postoperative complications are less likely to report satisfaction with their surgical outcomes and are more prone to decisional regret, diminished quality of life, and poorer psychosocial adjustment [27]. These consequences underscore the broader importance of minimizing postoperative complications, not only to improve clinical outcomes but also to enhance patient well-being and optimize the cost-effectiveness of care. In the context of nerve interface procedures such as TMR and RPNIs, reducing wound-related complications is particularly critical, as these can compromise the integrity of nerve repairs, delay prosthetic rehabilitation, and undermine the long-term benefits of pain reduction and improved function that these techniques aim to achieve.

It is well established that urgent or emergent surgeries carry a higher risk of postoperative complications compared with elective procedures [28]. Given that a substantial proportion of patients undergoing acute TMR/RPNIs present with active infection and greater medical complexity at the time of amputation, caution is warranted when assessing whether acute nerve interface surgery confers a net advantage in this subgroup. Careful patient selection and individualized timing are therefore critical to optimizing outcomes. Although numerous studies have reported that acute TMR and RPNI procedures are associated with lower pain scores and reduced neuroma recurrence [3,15,29,30], our findings provide a complementary perspective: higher observed rates of surgical complications were noted among patients undergoing acute procedures, likely reflecting differences in baseline risk and clinical context rather than the independent effect of timing alone.

These findings suggest that while acute nerve interface reconstruction may offer meaningful benefits in pain control and neuroma prevention, it may also carry greater perioperative risks in medically fragile patients. In the acute trauma setting, factors such as tissue contamination, ischemia, and hemodynamic instability may adversely affect healing and increase complication rates [31]. In contrast, delayed procedures performed in a controlled, well-healed surgical environment may yield more predictable outcomes and reduce the risk of wound-related complications, particularly when performed away from compromised distal soft tissues. These considerations underscore the importance of personalized surgical planning and thorough preoperative counseling, particularly for patients with acute infections, poor baseline health, or multiple comorbidities, who inherently face a higher risk of postoperative complications [32].

Beyond pain control, major lower-extremity amputation is well recognized to have a profound impact on overall quality of life, affecting physical mobility, social participation, and psychological well-being. Systematic reviews have demonstrated that individuals with lower limb amputations consistently report reduced quality-of-life outcomes compared with population norms, with pain, impaired mobility, and difficulty with prosthetic use serving as key determinants of diminished quality of life [33]. Chronic residual limb pain and phantom limb pain have been shown to be among the strongest predictors of poor quality-of-life outcomes in amputee populations [34]. Interventions such as targeted muscle reinnervation and regenerative peripheral nerve interfaces, which effectively reduce neuropathic pain and neuroma formation, therefore have the potential to improve quality-of-life outcomes indirectly by facilitating improved prosthetic tolerance, functional mobility, and participation in daily activities [18]. While quality-of-life measures were not directly assessed in the present study, existing literature supports the clinical relevance of TMR and RPNI in addressing major drivers of reduced quality of life following lower-extremity amputation.

To our knowledge, this is the first study to investigate acute and delayed TMR/RPNIs with respect to complication and reoperation rates in a cohort consisting solely of lower-extremity amputees. A study limited to lower-extremity amputees provides a more accurate assessment of outcomes associated with TMR and RPNIs, as upper and lower limb amputations present distinct anatomical, physiological, and rehabilitative considerations. Factors such as greater soft-tissue tension, and weight-bearing requirements in the lower limb can substantially alter wound healing dynamics and complication profiles, which may not be captured in mixed-cohort studies [35].

Nonetheless, this study has several limitations. As a retrospective cohort analysis, it is inherently subject to unmeasured confounding, including confounding by indication, whereby patients undergoing acute TMR/RPNIs more frequently presented with infection, ischemia, or greater medical complexity. Although multivariable logistic regression was performed to adjust for clinically relevant confounders, residual confounding from factors not captured in the dataset may still have influenced the results. The single-institution design and modest sample size further limit the generalizability of the findings. Patients undergoing acute TMR/RPNIs represent a broader cohort, many of whom may not have developed symptomatic neuromas in the absence of prophylactic intervention. Accordingly, differences in baseline risk profiles and amputation etiology should be carefully considered when interpreting comparative outcomes across timing groups. Definitions of ‘acute’ intervention in this context are necessarily pragmatic and informed by clinical practice, and future studies with larger cohorts may permit more biologically granular classification of surgical timing.

Additionally, although the odds ratios suggested potentially clinically meaningful differences between acute and delayed procedures, the wide confidence intervals reflect imprecision in the effect estimates. This imprecision is primarily due to the small number of complication events, which limits statistical power and increases the risk of both type II error and model overfitting in multivariable analyses. As such, the lack of statistical significance should not be interpreted as evidence of no association. Future multicenter studies with larger cohorts, prospective designs, and standardized operative and follow-up protocols are needed to validate these findings and better define patient selection criteria and optimal timing of nerve interface reconstruction in lower-extremity amputees.

## 5. Conclusions

TMR and RPNIs are effective surgical techniques that have been proven to improve both PLP and RLP in lower-extremity amputees. While acute TMR/RPNI procedures may be associated with a higher observed risk of surgery-related complications in more medically complex patients compared with delayed interventions, these procedures offer meaningful benefits in neuroma prevention, pain reduction, and functional rehabilitation that may outweigh the risks in appropriately selected patients. In this study, our findings highlight the importance of careful patient selection, optimized surgical timing, and individualized perioperative planning, particularly in patients with complex comorbidities or compromised soft-tissue environments.

## Figures and Tables

**Table 1:** Comparison of demographics between patients with acute and delayed TMR/RPNI.

	Acute TMR/RPNI (%)	Delayed TMR/RPNI (%)	*p* Value
No. total patients	45	29	
Male sex	31/45 (68.9)	18/29 (62.1)	0.5476
Mean age ± SD, yr	57.5 ± 17.4	52.1 ± 19.7	0.2253
Body Mass Index ± SD, kg/m^2^	26.3 ± 6.0	26.1 ± 6.1	0.8928
Comorbidities			
Hypertension	26/45 (57.8)	9/29 (31.0)	0.0255
Dyslipidemia	16/45 (35.6)	8/29 (27.6)	0.4777
Coronary Artery Disease	8/45 (17.8)	4/29 (13.8)	0.6521
Peripheral Vascular Disease	11/45 (24.4)	6/29 (20.7)	0.7097
Diabetes Mellitus	19/45 (42.2)	5/29 (17.2)	0.0260
Chronic Kidney Disease	6/45 (13.3)	4/29 (13.8)	0.9553
Cancer	5/45 (11.1)	0/29 (0)	0.0648
Smoking History	25/45 (55.6)	15/29 (51.7)	0.7485
No. total limbs	47	33	
Reason for amputation			0.0076
Infection	17 (36.2)	2 (6.1)	
Ischemia	15 (31.9)	10 (30.3)	
Trauma	9 (19.1)	16 (48.5)	
Cancer	3 (6.4)	1 (3)	
Other	3 (6.4)	4 (12.1)	
Level of Amputation			0.0002
Below-knee/transtibial/calf	41 (87.2)	16 (48.5)	
Above-knee/transfemoral/thigh	6 (12.8)	17 (51.5)	
No. of nerves addressed			0.1615
N/A	4 (8.5)	0 (0)	
1	5 (10.6)	3 (9.1)	
2	15 (31.9)	19 (57.6)	
3	15 (31.9)	6 (18.2)	
4	3 (6.4)	3 (9.1)	
5	5 (10.6)	2 (6.1)	

Abbreviations: TMR, targeted muscle reinnervation; RPNI, regenerative peripheral nerve interface.

**Table 2:** Comparison of procedures performed between patients with acute and delayed TMR/RPNI.

	Acute TMR/RPNI (%)	Delayed TMR/RPNI (%)	*p* Value
Total cases	45	29	
Total limbs	47	33	
Procedures performed			0.0716
TMR only	29 (61.7)	20 (60.6)	
RPNI only	5 (10.6)	9 (27.3)	
TMR and RPNI	13 (27.7)	4 (12.1)	

Abbreviations: TMR, targeted muscle reinnervation; RPNI, regenerative peripheral nerve interface.

**Table 3:** Comparison of complications between patients with acute and delayed TMR/RPNI.

	Acute TMR/RPNI (%)	Delayed TMR/RPNI (%)	*p* Value
Total limbs	47	33	
Total complications	11	4	
Complication rate	11/47 (23.4)	4/33 (12.1)	0.2059
Surgical complication rate	11/47 (23.4)	2/33 (6.1)	0.0397
Hematoma	1 (9.1)	0 (0)	
Seroma	0 (0)	0 (0)	
Wound infection	3 (27.3)	2 (50)	
Non-healing wound	7 (63.6)	0 (0)	
Scar contracture	0 (0)	0 (0)	
Neuromas with pain	0 (0)	2 (50)	

Abbreviations: TMR, targeted muscle reinnervation; RPNI, regenerative peripheral nerve interface.

**Table 4:** Surgical complication rates stratified by amputation etiology between patients with acute and delayed TMR/RPNI.

	Acute TMR/RPNI Complications/Total (%)	Delayed TMR/RPNI Complications/Total (%)
Infection/Vascular	9/32	2/12
Trauma	2/9	0/16
Others	0/6	0/5

Abbreviations: TMR, targeted muscle reinnervation; RPNI, regenerative peripheral nerve interface.

**Table 5:** Comparison of reoperations between patients with acute and delayed TMR/RPNI.

	Acute TMR/RPNI (%)	Delayed TMR/RPNI (%)	*p* Value
Total limbs	47	33	
Total reoperations	11	7	
Total reoperation rate	11/47 (23.4)	7/33 (21.2)	0.8183
Unexpected reoperation rate	9/47 (19.1)	4/33 (12.1)	0.4045
Hematoma	1 (9.1)	0 (0)	
Seroma	0 (0)	0 (0)	
Wound infection	1 (9.1)	2 (28.6)	
Non-healing wound	7 (63.6)	0 (0)	
Scar contracture	0 (0)	0 (0)	
Neuromas with pain	0 (0)	2 (28.6)	
Expected reoperations	2	3	
Staged osseointegration	2 (18.2)	3 (42.9)	

Abbreviations: TMR, targeted muscle reinnervation; RPNI, regenerative peripheral nerve interface.

**Table 6:** Univariable logistic regression evaluating predictors of surgical complications following acute vs. delayed TMR/RPNI.

	OR	95% CI Lower	95% CI Upper	*p* Value
Acute TMR/RPNI	4.7361	0.9742	23.0247	0.0539
Age at Surgery	1.055	1.0137	1.0981	0.0087
Sex	0.7822	0.229	2.6714	0.6951
Body Mass Index	0.9487	0.8428	1.0679	0.3832
Smoking History	2.0571	0.5768	7.3372	0.2662
Hypertension	8.6731	1.7779	42.3086	0.0075
Dyslipidemia	4.3556	1.259	15.0678	0.0201
Coronary Artery Disease	0.9256	0.1796	4.7692	0.9264
Peripheral Vascular Disease	4.8857	1.3572	17.5874	0.0152
Diabetes Mellitus	2.0143	0.601	6.7515	0.2565
Chronic Kidney Disease	2.2125	0.5003	9.7838	0.2951
Cancer	1.3125	0.1347	12.7889	0.8149

Abbreviations: OR, odds ratio; CI, confidence interval.

**Table 7:** Multivariable logistic regression evaluating independent predictors of surgical complications following acute vs. delayed TMR/RPNI.

	OR	95% CI Lower	95% CI Upper	*p* Value
Acute TMR/RPNI	4.3209	0.725	25.7531	0.1081
Age at Surgery	1.0336	0.9865	1.0829	0.1646
Sex	0.5376	0.1126	2.5671	0.4366
Body Mass Index	0.9016	0.7684	1.0578	0.2038
Smoking History	0.9657	0.1579	5.9061	0.9698
Hypertension	4.6773	0.5688	38.4637	0.1513
Dyslipidemia	1.7731	0.3129	10.0479	0.5176
Peripheral Vascular Disease	1.9597	0.4087	9.3965	0.4002

Abbreviations: OR, odds ratio; CI, confidence interval.

## Data Availability

The data analyzed in the current study are available from the corresponding author upon reasonable request.

## Abbreviations

The following abbreviations are used in this manuscript:

TMR	Targeted Muscle Reinnervation
RPNIs	Regenerative Peripheral Nerve Interfaces
